# Supplementary material for: Development and Validation of a Highly Sensitive LC-MS/MS Method for the Analysis of Bile Acids in Serum, Plasma, and Liver Tissue Samples
Source: Metabolites. 2020 Jul 9;10(7):282. doi: 10.3390/metabo10070282 (PMC7408441; doi:10.3390/metabo10070282)
Supplement: Supplementary file 1 [file metabolites-10-00282-s001.pdf]

Supplementary file

Development and validation of a highly sensitive LC-MS/MS method for the analysis of bile acids in serum, plasma and liver tissue samples

Cristina Gómez[1], Simon Stücheli[1], Denise V. Kratschmar[1], Jamal Bouitbir[1], Alex Odermatt[1]

[1] Division of Molecular and Systems Toxicology, Department of Pharmaceutical Sciences, University of Basel, Klingelbergstrasse 50, 4056 Basel, Switzerland.

Supplementary materials and methods

Lipid profile analysis

Total cholesterol, HDLc, and LDLc/VLDLc were measured by colorimetric assay according to the manufacturer's protocol using HDL and LDL/VLDL cholesterol assay kit (Abcam, ab 65390, Cambridge, England, UK).

Supplementary results

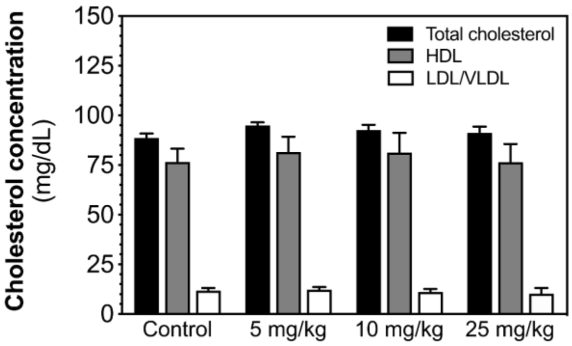

Supplementary figure 1*:* Lipid profile measured in simvastatin-treated and control mice. The graph shows the concentrations of total cholesterol, HDLc and LDLc/VLDLc in control and simvastatin-treated mice for three weeks at 5, 10, and 25 mg/kg. Data are expressed as mean ± SEM of six independent experiments.

Table S1: Concentrations of individual bile acids quantified in serum, plasma and liver tissue from mice and in human plasma. Values are expressed as mean ± SD (nM for serum and plasma, pg/mg tissue for liver samples). ND, not detected.

Name	Mouse serum n=9 (nM)	Mouse plasma n=6 (nM)	Mouse liver n= 20 (pg/mg tissue)	Human plasma n=18 (nM)
**CA**	289 ± 187	712 ± 368	1264 ± 1918	27 ± 77
**CDCA**	17 ± 12	58 ± 12	105 ± 102	63 ± 99
**ωMCA**	208 ± 129	194 ± 70	891 ± 702	ND
**αMCA**	53 ± 30	57 ± 15	446 ± 681	ND
**βMCA**	281 ± 171	609 ± 388	5384 ± 6344	ND
**γMCA**	ND	ND	ND	ND
**UDCA**	7 ± 8	193 ± 97	47 ± 56	1 ± 2
**HDCA**	ND	95 ± 57	49 ± 14	ND
**DCA**	220 ± 140	278 ± 78	70 ± 14	91 ± 80
**LCA**	ND	ND	ND	ND
**3oxoLCA**	ND	ND	ND	ND
**7oxoLCA**	2 ± 2	17 ± 19	4 ± 10	0,5 ± 0,8
**12oxoLCA**	6 ± 5	ND	20 ± 28	1,4 ± 1
**6,7dioxoLCA**	ND	ND	107 ± 89	ND
**7oxoDCA**	221 ± 141	149 ± 39	472 ± 1171	ND
**Allo-CA**	73 ± 46	40 ± 15	172 ± 180	ND

**Allo-DCA**	27 ± 20	24 ± 26	ND	0,04 ± 0,1
**Allo-3βDCA**	1 ± 2	ND	ND	6 ± 6
**Allo-12βDCA**	ND	ND	ND	ND
**Allo-3βLCA**	ND	ND	ND	ND
**Allo-LCA**	ND	ND	ND	ND
**TCA**	16160 ± 6702	12006 ± 6995	156284 ± 122604	14 ± 10
**TCDCA**	1104 ± 598	441 ± 37	7060 ± 14945	50 ± 41
**TωMCA**	5369 ± 2737	1902 ± 364	57347 ± 64084	ND
**TαMCA**	4538 ± 2336	2280 ± 447	33327 ± 56545	ND
**TβMCA**	9917 ± 4008	8705 ± 5520	138231 ± 137542	ND
**TUDCA**	1330 ± 657	645 ± 116	11643 ± 18165	6 ± 7
**TDCA**	839 ± 508	535 ± 49	7532 ± 12444	19 ± 13
**T7oxoLCA**	242 ± 141	83 ± 13	2268 ± 3185	0,03 ± 0,1
**TLCA**	18 ± 15	27 ± 29	469 ± 115	0,9 ± 1
**GCA**	81 ± 45	38 ± 8	885 ± 1868	51 ± 37
**GCDCA**	3 ± 1	9 ± 1	17 ± 13	226 ± 191
**GUDCA**	5 ± 2	3 ± 1	15 ± 15	65 ± 80
**GDCA**	ND	ND	13 ± 8	71 ± 48
**GLCA**	ND	ND	ND	9 ± 7
**G7oxoLCA**	ND	ND	ND	ND

Table S2: Concentrations of individual bile acids quantified in plasma and liver tissue in simvastatin-treated and control mice. Values are expressed as mean ± SD (nM for plasma, pg/mg tissue for liver samples). ND, not detected.

Name	Mouse plasma n=24 (nM)	Mouse liver n= 24 (pg/mg tissue)
**CA**	125 ± 77	284 ± 248
**CDCA**	21 ± 12	6 ± 4
**ωMCA**	314 ± 213	113 ± 91
**α-MCA**	15 ± 11	80 ± 74
**βMCA**	213 ± 136	668 ± 532
**γMCA**	ND	ND
**UDCA**	36 ± 23	13 ± 11
**HDCA**	15 ± 11	12 ± 7
**DCA**	247 ± 130	21 ± 13
**LCA**	ND	ND
**3oxoLCA**	ND	ND
**7oxoLCA**	ND	ND
**12oxoLCA**	4 ± 2	3 ± 4
**6,7dioxoLCA**	ND	ND
**7oxoDCA**	96 ± 100	292 ± 354
**Allo-CA**	15 ± 12	23 ± 16
**Allo-DCA**	16 ± 8	5 ± 2
**Allo-3βDCA**	ND	3 ± 1

**Allo-12βDCA**	ND	ND
**Allo-3βLCA**	ND	ND
**Allo-LCA**	ND	ND
**TCA**	528 ± 952	20521 ± 15487
**TCDCA**	11 ± 13	1195 ± 562
**TωMCA**	251 ± 388	10759 ± 8692
**TαMCA**	154 ± 263	4921 ± 3858
**TβMCA**	381 ± 713	20036 ± 13613
**TUDCA**	26 ± 29	1709 ± 793
**TDCA**	65 ± 48	4000 ± 1865
**T7oxoLCA**	4 ± 7	185 ± 123
**TLCA**	ND	191 ± 89
**GCA**	7 ± 4	59 ± 45
**GCDCA**	ND	ND
**GUDCA**	ND	ND
**GDCA**	ND	ND
**GLCA**	ND	ND
**G7oxoLCA**	ND	ND
